# TWIST1, A novel androgen-regulated gene, is a target for NKX3-1 in prostate cancer cells

**DOI:** 10.1186/1475-2867-13-4

**Published:** 2013-01-31

**Authors:** Turid Eide, Håkon Ramberg, Carlotta Glackin, Donald Tindall, Kristin Austlid Taskén

**Affiliations:** 1 Department of Tumor Biology, Institute for Cancer Research, Norwegian Radium Hospital, Oslo University Hospital, Oslo, Norway; 2 Faculty of Medicine, University of Oslo, Oslo, Norway; 3 Division of Molecular Medicine, Beckman Research Institute of the City of Hope, Duarte, 91010-3011, CA, USA; 4 Department of Urology, Mayo Clinic and Foundation, Rochester, 55905, MN, USA

**Keywords:** Prostate cancer, LNCaP cells, TWIST1, NKX3-1

## Abstract

**Background:**

TWIST1 plays a key role in EMT-mediated tumor invasion and metastasis. Since bone metastasis is a hallmark of advanced prostate cancer and is detected in at least 85% of patients who die of this disease, it is of great importance to understand the regulation of the cellular signaling pathways involved in the metastatic process.

**Methods:**

Prostatic cell lines were analyzed using real time RT-PCR, chromatin immunoprecipitations (ChIP) and transfection of siRNA’s and reporter constructs.

**Results:**

We report in this paper that TWIST1 is an androgen-regulated gene under tight regulation of NKX3-1. Androgens repress the expression of TWIST1 via NKX3-1, which is a prostate–specific tumor suppressor that is down-regulated in the majority of metastatic prostate tumors. We show that NKX3-1 binds to the TWIST1 promoter and that NKX3-1 over-expression reduces the activity of a TWIST1 promoter reporter construct, whereas NKX3-1 siRNA up-regulates endogenous TWIST1 mRNA in prostate cancer cells.

**Conclusion:**

Our finding that NKX3-1 represses TWIST1 expression emphasizes the functional importance of NKX3-1 in regulating TWIST1 expression during prostate cancer progression to metastatic disease.

## Background

Metastasis is the leading cause of morbidity and mortality among men with prostate cancer (PCa). Metastasis is a complex multistep process controlled by distinct genes and signaling pathways in each step. Epithelial-meschencymal transition (EMT), a critical event for morphogenetic movements during formation of parietal endoderm during gastrulation, may represent the initial phase of metastasis. Furthermore, EMT seems to induce stem-like properties of epithelial cells [1].

A pivotal step of EMT is the loss of E-cadherin [2]. TWIST1, a master regulator of mesodermal development and a key mediator in the metastatic process, represses the expression of E-cadherin [3]. Furthermore, TWIST1 depletion reduces the expression of N-cadherin in PC3 cells, a metastatic prostate cancer cell line, suggesting that TWIST1 supports EMT in prostate cancer [4]. In support of this, a positive correlation between the level of TWIST1 and prostate cancer metastasis has been reported [5].

NKX3-1 is a critical gene associated with early stage of prostate tumorigenesis as down-regulation of NKX3-1 is observed in both prostatic intraepithelial neoplasia (PIN) and adenocarsinomas [6]. Whereas high levels of TWIST1 is expressed in prostate cancer metastasis, low levels of NKX3-1 expression is observed in most prostate cancer metastasis examined [7]. NKX3-1 encodes a homeobox gene that is switched on during embryonic development of prostate tissue and is one of the earliest markers of luminal prostate epithelium [8]. NKX3-1 expression is strictly regulated by androgens and also appears to mark a sub-population of prostate stem cells [9,10].

We report in this paper that TWIST1 is a novel androgen-regulated gene whose expression is tightly controlled by NKX3-1.

## Results

### TWIST1 is regulated by androgens

**Figure 1:**
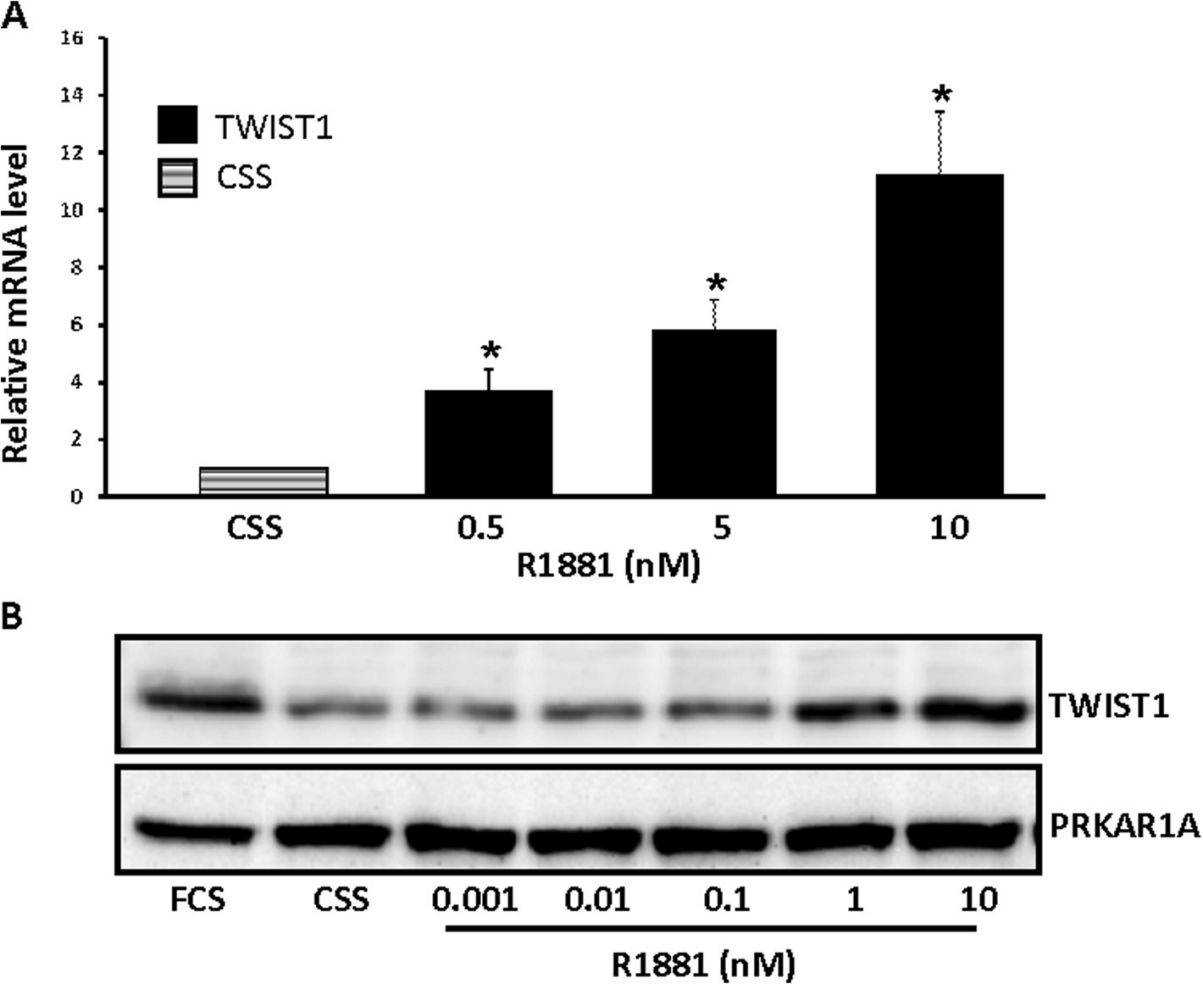
**Androgen up-regulates TWIST1 expression.A**. LNCaP cells were stimulated with increasing doses of the synthetic androgen R1881, and the relative level of TWIST1 compared to LNCaP cells left untreated in charcoal stripped fetal calf serum and phenol red free medium (CSS) was quantified using sqRT-PCR. Data are presented as mean ± SD (n=3) relative to unstimulated cells (control). A paired t-test was performed and a two-tailed p-value <0,05 is indicated with a *. **B**. LNCaP cells were stimulated with increasing doses of the synthetic androgen R1881 or left untreated in CSS for 3 days before total protein was extracted. A representative Western blot probed with anti human TWIST1 antibody and anti PRKAR1A antibody as loading control is shown.

The expression of TWIST1 mRNA increased 4-fold in LNCaP cells incubated with 0.5 nM R1881 for 72 hours relative to untreated cells (ctr.) and reached more than a 10-fold induction at 10 nM R1881 (Figure 1A). Corresponding regulation of TWIST1 mRNA was also demonstrated in RWPE-1, a benign prostate cell line (data not shown). Up-regulation of TWIST1 expression was also observed at the protein level in LNCaP cells stimulated with increasing concentrations of R1881 (Figure 1B).

**Figure 2:**
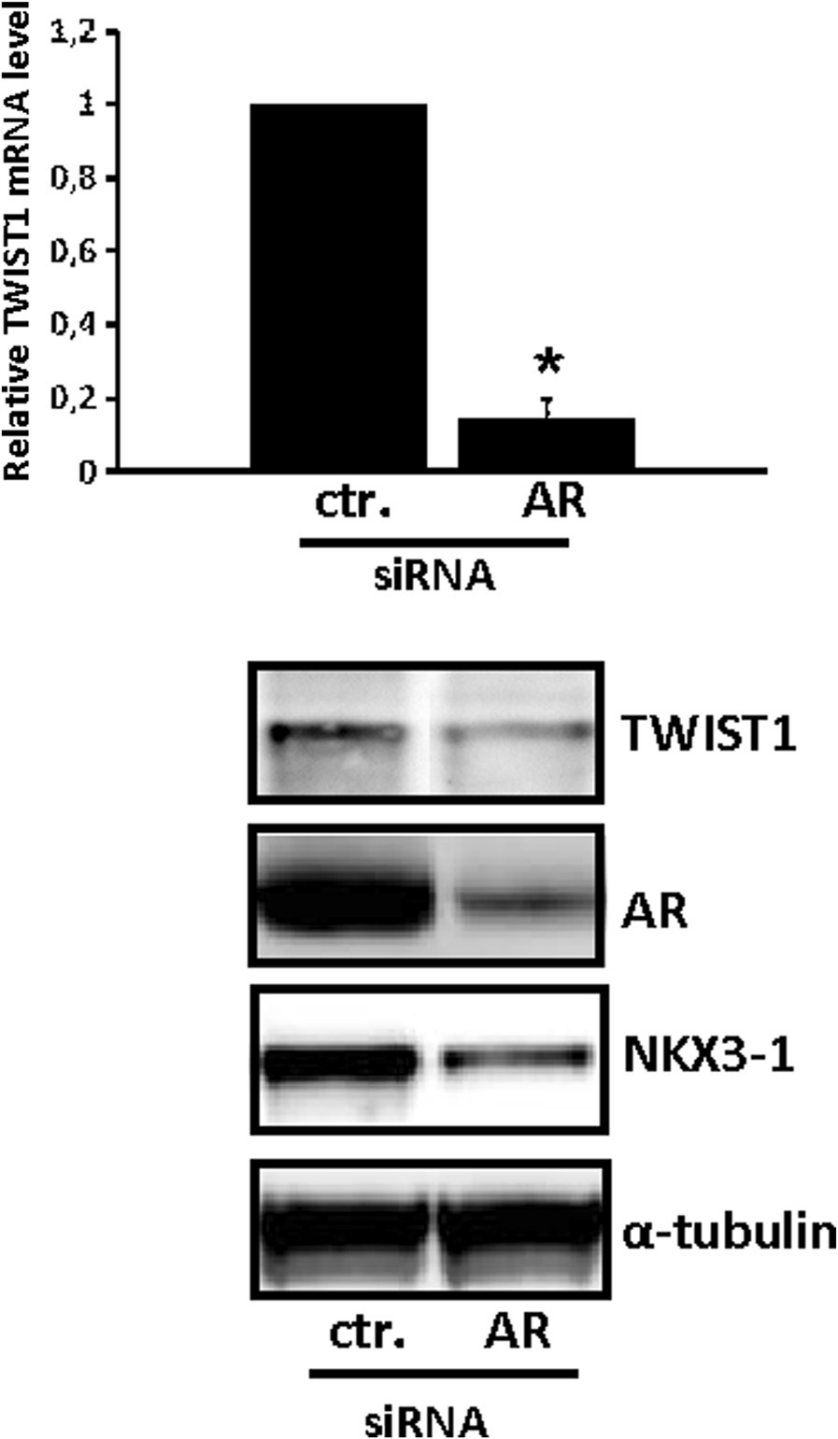
**AR mediates the effect of androgen on TWIST1 expression.** LNCaP cells cultured in RPMI medium containing 10% FCS were transfected with 100 nM of siRNA against AR (AR) or a non-silencing control (ctr.) siRNA. Total RNA and protein was harvested at 48 h after transfection. **Upper panel**: TWIST1 mRNA expression was examined using sqRT-PCR. Data are presented as mean ± SD (n=3) relative to the levels of LNCaP cells transfected with a non-silencing control siRNA. A paired t-test was performed and a two-tailed p value <0,05 is indicated with a *. **Lower panel:** Representative Western blots of TWIST1, AR, NKX3-1 and α**-**tubulin (loading control) are shown.

To determine if AR mediates the effect of androgens on TWIST1 expression, LNCaP cells were transfected with a siRNA targeting AR (AR) or a non-silencing siRNA as control (ctr.). Down-regulation of AR expression (Figure 2, lower panel) reduced the mRNA level of TWIST1 by 80% relative to control (Figure 2A, upper panel) supporting the concept that TWIST1 is an AR target gene in LNCaP cells. Down-regulation of AR also reduced the level of NKX3-1 (Figure 2, lower panel).

### TWIST1 is an NKX3-1 target gene

**Figure 3:**
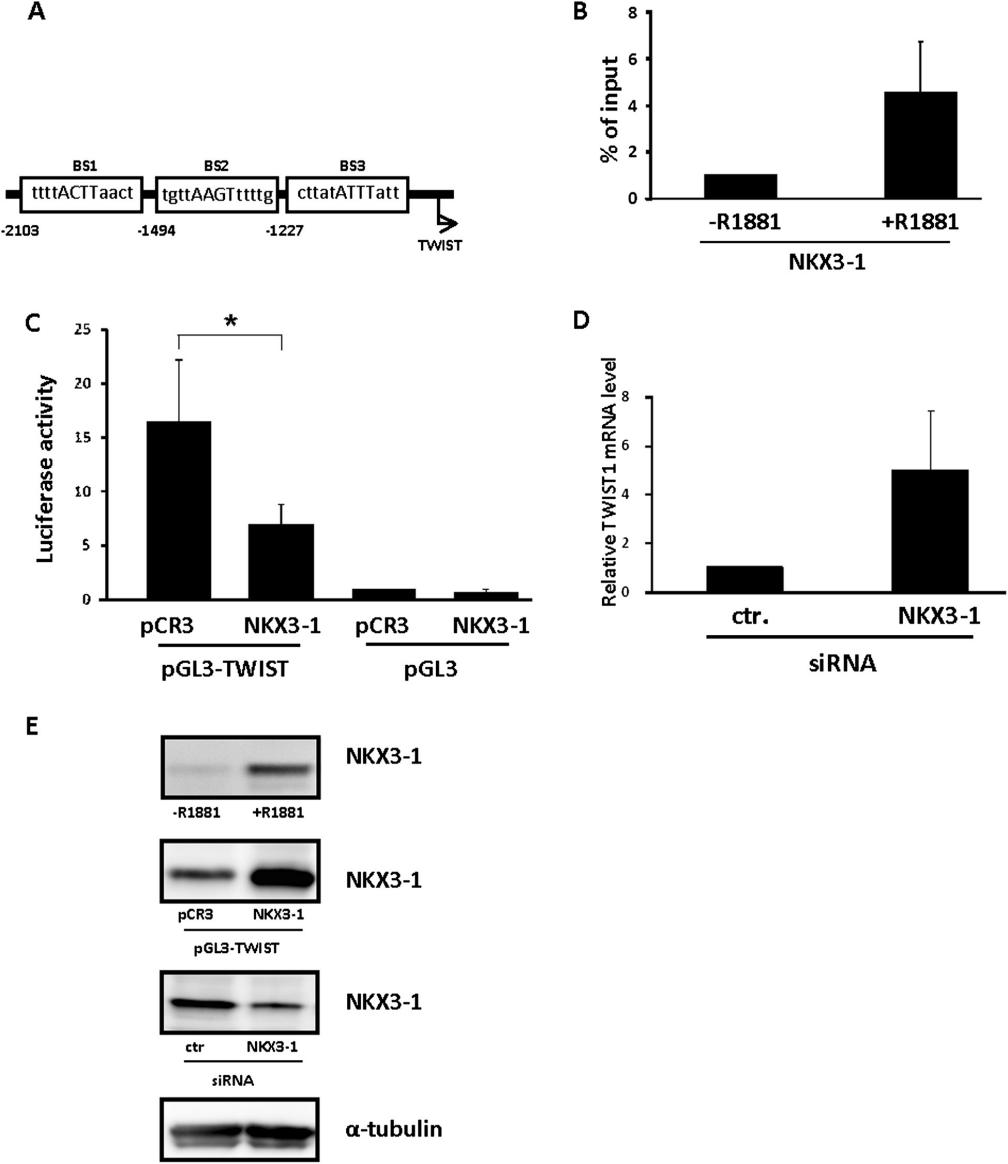
**NKX3-1 mediates androgen-regulation of TWIST1. A**. Three putative NKX3-1-bindingsites (BS1, BS2 and BS3) are shown as boxes in the TWIST1 promoter (from NCBI MapViewer). **B**. Chromatin immunoprecipitation was performed using chromatin isolated from LNCaP cells stimulate with 10 nM R1881 (+R1881) for 24 hours or left untreated (−R1881) and using a monoclonal antibody against NKX3-1 (NKX3-1). sqPCR results using primers targeting BS3 are shown in B. The results of the BS3 primer set are shown adjusted to the levels of chromatin immunoprecipitated with IgG as a negative control. Data are presented as mean ± SD (n=3). **C**. LNCaP cells were transfected with pGL3-TWIST-Luc (pGL3-TWIST) and a human NKX3-1 expression plasmid or an empty vector (pCR3) as well as pCMV β-gal. As control, LNCaP cells were transfected with a reporter plasmid lacking the TWIST promoter region (pGL3) and the human NKX3-1 expression vector or an empty expression plasmid (pCR3). The luciferase activities were normalized against the corresponding β-galactosidase activities and the results are shown as mean ± SD (n=3). A paired t-test was performed and a two-tailed p value <0,05 is indicated with a * **D**. LNCaP cells were transfected with 100 nM of siRNA targeting NKX3-1 or a non-silencing siRNA (ctr.) and total RNA was harvested 72 hours after transfection. TWIST1 mRNA expression was examined using sqRT-PCR. The data are shown as mean ± SD (n=3) relative to the levels of LNCaP cells transfected with a non-silencing control siRNA. **E**. Representative Western blots showing expression levels of NKX3-1 in (from top) LNCaP cells stimulated with 10 nM of R1881, transfected with NKX3-1 overexpression vector or targeted by siRNA against NKX3-1. α**-**tubulin is shown as loading control.

In an effort to find binding sites for AR (ARE) or androgen-regulated transcription factors, we examined the human TWIST1 genomic sequence using MatInspector [11]. Three putative binding sites for NKX3-1 designated BS1, BS2 and BS3 (Figure 3A) were identified. In addition we observed several potential AREs. Chromatin immunopreciptation analyses (ChIP) using chromatin isolated from LNCaP cells stimulate with 10 nM R1881 (+R1881) for 24 hours or unstimulated cells as control (−R1881) resulted in amplification of BS3. We were not able to detect binding to either BS1 or BS2 (data not shown, n=3). A 5-fold increase in NKX3-1 binding activity to the TWIST1 promoter upon R1881 stimulation was observed (Figure 3B). Additionally, PCR amplification of non-targeted DNA (genomic contig of chromosome 12) was used as negative control (data not shown).

Next, a reporter construct containing 5kb of the mouse TWIST1 promoter (pGL3-TWIST-Luc) was co-transfected with an NKX3-1 expression vector. Over-expression of NKX3-1 reduced the promoter activity of TWIST1 by 50%, indicating that NKX3-1 represses the TWIST1 promoter activity (Figure 3C). No effect of NKX3-1 over-expression was observed on cells transfected with pGL3 lacking the TWIST1 promoter (pGL3).

Finally, the effect of NKX3-1 on endogenous TWIST1 mRNA level was studied by transfecting LNCaP cells with siRNA targeting NKX3-1. A 5-fold increase in TWIST1 mRNA level was observed when NKX3-1 expression was down-regulated (Figure 3D). The impact of R1881 stimulation, NKX3-1 overexpression and siRNA on NKX3-1 expression in LNCaP cells are summarized in Figure 3E. Both R1881 stimulation as well as overexpression of NKX3-1 led to a significant increase in NKX3-1 expression, while siRNA against NKX3-1 reduced the expression markedly.

## Discussion

In this study we show both that TWIST1 mRNA is up-regulated by androgen via AR and that NKX3-1, a well-known androgen-regulated gene, binds the upstream regulatory region of the TWIST1 gene and represses the expression of TWIST1.

In a recent study, Takayama et al. [12] identified putative direct target genes of AR using ChIP-on ChIP and Cap Analysis Gene Expression (CAGE). One of the genes identified by *in silico* analysis was TWIST1. Our results support this report as increased levels of TWIST1 mRNA was observed in R1881 stimulated LNCaP cells, while reduced expression of TWISTl was observed in cells transfected with siRNA targeting AR.

Furthermore, our data shows binding of NKX3-1 to the promoter region of TWIST1. We therefore suggest that NKX3-1 mediates an indirect and late effect of androgen stimulation on TWIST1 expression. Interestingly, we show that siRNA targeting AR reduces the level of TWIST1, whereas siRNA targeting NKX3-1 increases TWIST1 expression suggesting that TWIST1 expression is tightly controlled by androgen. NKX3-1 has been shown to function both as an activator and a repressor of transcription, but few target genes have been identified [13-15].

The physical binding of NKX3-1 to the TWIST1 promoter might block the mesenchymal drive of TWIST1, until NKX3-1 expression is down-regulated or lost in PIN or adenocarcinoma lesions. Loss of NKX3-1 expression has been observed in ~20% of PIN lesions, ~40% of advanced prostate tumors and up to 80% of metastatic prostate cancer [16]. Androgen deprivation therapy as the most widely used treatment for advanced prostate cancer is likely to abolish androgen-stimulation of NKX3-1, leading eventually to down-regulation of repressor protein and de-repression of TWIST1’s metastatic potential.

In an attempt to identify genes whose regulation are altered by NKX3-1, Song et al. [17] performed gene expression profiling analyses on micro dissected glands from NKX3-1-deficient prostate tissues during prostate cancer progression. They observed similarities between the expression profile of the micro dissected glands and constitutive activated AKT-transgenic mice as well as PTEN-deficient mice, suggesting that the PTEN-AKT-NKX3-1 axis serve as a major molecular path of prostate tumorigenesis. Li and Zhou [18] showed that activation of the AKT pathway by TWIST1 is critical for the sustention of cancer stem cell-like traits generated by EMT, again suggesting a link between loss of NKX3-1 expression, relive of TWIST1 expression and eventually activation of AKT pathway.

## Conclusions

We report in this paper that TWIST1 is an androgen-regulated gene, tightly regulated by NKX3-1. We show that NKX3-1 binds to the TWIST1 promoter and that NKX3-1 over-expression reduces the activity of a TWIST1 promoter reporter construct, whereas NKX3-1 siRNA up-regulated endogenous TWIST1 mRNA in prostate cancer cells. Our finding that NKX3-1 represses TWIST1 expression emphasizes the functional importance of NKX3-1 in regulating TWIST1 expression during prostate cancer progression to metastatic disease.

## Methods

### Cell culture

LNCaP and RWPE-1 cells were purchased from ATCC (Rockville, MD) and cultured in RPMI 1640 medium containing 10% fetal calf serum (FCS) or Keratinocyte-SFM medium from Invitrogen (Carlsbad, CA, USA) supplemented with 2.5 μg Epidermal Growth Factor (EGF) and 25 mg Bovine Pituitary Extract (BPE), respectively, and stimulation with synthetic androgen R1881 was performed as previously described [19].

### Semi-quantitative real time RT-PCR (sqRT-PCR)

Total RNA was isolated using Trizol™ from Invitrogen (Carlsbad, CA), and 100 ng of total RNA was used in a one-step RT-PCR reaction (QIAGEN Quantitect SYBR Green RT-PCR kit) that was performed using an MJ Research DNA Engine Opticon Continuous Fluorescence Detection System (MJ Research Inc., Waltham, MA). RT-PCR cycles were performed as previously described by Ramberg et al. [20]. G6PD was used for normalization. The ΔΔCt formula was used as described in the protocol from Applied Biosystems (Foster City, CA). All the PCR-products were verified by sequencing.

### Primer sets used in sqRT-PCR

G6PD (NM_000402); Left: tgcatgagccagataggc and right: acagggaggagatgtggttg, NKX3-1 (NM_006167); Left: gagacgctggcagagacc and right: ttctgcggctgcttaggg, AR (NM_000044); Left: gcgatccttcaccaatgtca and right: cattcggacacactggctgt, TWIST1 (NM_000474); Left: cttctcggtctggaggatgg and right: ctccttctctggaaacaatgaca.

### Immunoblotting

Protein extraction followed by Western blot analysis was performed as previously described by Kvissel et al. [19]. Primary antibodies used were the following: anti-TWIST1 (H-81, sc-15393), anti-AR (N-20, sc-816), both from Santa Cruz Biotechnology (Santa Cruz, CA). The anti-NKX3-1 antibody was kindly provided by Professor Fahri Saatcioglu at Department of Molecular Biosciences, University of Oslo, Norway. For loading control we used anti-PRKAR1A (610609, BD Transduction Laboratories) or anti-α-tubulin antibody from Sigma (St. Louis, MO).

### Transfection and luciferase assay

LNCaP cells were cultured at 300.000 cells per 6-well dish and transfected with a luciferase reporter plasmid including 5 kb of the mouse TWIST1 promoter- pGL3-TWIST (kindly provided by Steven Kendall and Carlotta Glackin, Beckman Research Institute, City of Hope, USA) or pGL3 as negative control using Lipofectamine 2000 (INVT11668019, Invitrogen) according to the manufactures protocol. For overexpression of NKX3-1, a commercial transfection-ready TRUE clone (sc116287, ORIGENE, Rockville, MD, USA) was purchased. Cells were co-transfected with pCMV β-gal (Clontech) to monitor the transfection efficiency. After 72 hours, luciferase and β-galactosidase activity were measured as previously described [20].

### Preparation and transfection of synthetic small interfering RNA (siRNA)

siRNA targeting the following sequence within the androgen receptor mRNA: 5’-AAAAGCCCATCGTAGAGGCCCCA-3’ was purchased from Dharmacon (Dharmacon Inc. (Lafayette, CO). A non-silencing siRNA (cat.no D-001810-10-20, Dharmacon) was included in all siRNA experiments. siRNA against NKX3-1 was purchased from QIAGEN (2-for-silencing) and included the following sequences: NKX3-1-1: 5’-CAGGCTATCATATATACTGTA-3’, NKX3-1-2: 5’-ACGCTATAAGACTAAGCGAAA-3’. All siRNAs were transfected into cells using Dharma*FECT* ™ 3 transfection reagent according to the manufacture’s protocol (cat.no T-2003, Dharmacon).

### Chromatin immunoprecipitation

ChIP assays were carried out according to the QuickChIP protocol (Imgenex, San Diego, CA) with a crosslinking time of 10 minutes using 1% formaldehyde for the LNCaP cells and using an anti human NKX3-1 monoclonal antibody (cat.no 35–9700, Zymed Laboratories Inc., San Fransisco, CA). Following DNA purification (QuickChIP DNA Purification kit), sqPCR was performed using QIAGEN Quantitect SYBR Green RT-PCR kit with the following primers:

NKX3-1 BS3; Left: cccagttacacttggatgcagta and right: tcccctggtgagatcatacatac. Negative control primers from human chromosome 12 genomic contig; Left: atggttgccactggggatct and right: tgccaaagcctaggggaaga. Chromatin immunoprecipitated with IgG was used as a negative control. The ΔΔCt formula was used as described in the protocol from Applied Biosystems (Foster City, CA).

## Abbrevations

PCa: Prostate Cancer; EMT: Epithelial-Meschencymal Transition; PIN: Prostatic Intraepithelial Neoplasia; AR: Androgen Receptor; ChIP: Chromatin Immunopreciptation Analyses.

## Competing interests

The authors declare that they have no competing interest.

## Authors’ contributions

TE took part in designing the study and conducted the experiments, collected and analyzed the data, and wrote the manuscript. HR conducted the Western blot analysis of androgen stimulation of TWIST1 and took part in reviewing of the manuscript. CG contributed with the mouse TWIST1-promoter plasmid and took part in reviewing of the manuscript. DJT and KAT both conceived and coordinated the study, and participated in the discussion of all analyses and critical revision of the manuscript. All authors have read and approved the final draft of the manuscript.
